# Macrophages as Emerging Key Players in Mitochondrial Transfers

**DOI:** 10.3389/fcell.2021.747377

**Published:** 2021-10-13

**Authors:** Yidan Pang, Changqing Zhang, Junjie Gao

**Affiliations:** Department of Orthopaedic Surgery, Shanghai Jiao Tong University Affiliated Sixth People’s Hospital, Shanghai, China

**Keywords:** macrophage, mitochondrial transfer, mitophagy, adipocyte, cardiomyocyte

## Abstract

Macrophages are a group of heterogeneous cells widely present throughout the body. Under the influence of their specific environments, via both contact and noncontact signals, macrophages integrate into host tissues and contribute to their development and the functions of their constituent cells. Mitochondria are essential organelles that perform intercellular transfers to regulate cell homeostasis. Our review focuses on newly discovered roles of mitochondrial transfers between macrophages and surrounding cells and summarizes emerging functions of macrophages in transmitophagy, metabolic regulation, and immune defense. We also discuss the negative influence of mitochondrial transfers on macrophages, as well as current therapies targeting mitochondria in macrophages. Regulation of macrophages through mitochondrial transfers between macrophages and their surrounding cells is a promising therapy for various diseases, including cardiovascular diseases, inflammatory diseases, obesity, and cancer.

## Introduction

Macrophages, which were once considered to be supplied only by adult monocytes, are now known to have both bone marrow myeloid and embryonic origins (Ginhoux and Guilliams, 2016). In addition to their immune surveillance function, macrophages show plasticity according to their environment in different tissues; thus, they have tissue-specific roles in maintaining homeostasis and tight interactions with surrounding cells (Gosselin et al., 2014; Lavin et al., 2014; Okabe and Medzhitov, 2016). Aberrant differentiation, polarization, and functions of macrophages give rise to diseases in various systems (Wynn et al., 2013; Byrne et al., 2016; Krenkel and Tacke, 2017; Peet et al., 2020). Deficient generation of anti-inflammatory macrophages and failed communication between macrophages and epithelial cells, endothelial cells, fibroblasts, and stem or tissue progenitor cells all contribute to a state of persistent injury; this can lead to pathological fibrosis (Wynn and Vannella, 2016) such as that occurring in chronic lung disease (Kim et al., 2008; O’Beirne et al., 2020). In atherosclerosis, macrophages participate in a non-resolving inflammatory response that expands the subendothelial layer and results in thrombosis (Moore and Tabas, 2011). Tumor-associated macrophages (TAMs) in the tumor microenvironment are often associated with poor prognosis and chemoresistance and have thus recently emerged as therapeutic targets (Cassetta and Pollard, 2018; Xiang et al., 2021).

Mitochondria are vital organelles that constantly undergo inner cellular movements and intracellular transfers to fulfill energy needs and promote cell survival (Liesa et al., 2009; Youle and van der Bliek, 2012; Zampieri et al., 2021). Macrophages rely strongly on mitochondria for their activation and functions (Weinberg et al., 2015; Tur et al., 2017). Recent studies have identified various tissue-resident macrophages as important participants in intercellular mitochondrial transfers, unveiling a new function of such macrophages (Phinney et al., 2015; Brestoff et al., 2020; Nicolas-Avila et al., 2020). Macrophage-related mitochondrial transfers have critical roles in tissue homeostasis, metabolic regulation, and immune defense under both physiological and pathological conditions (Tables 1, 2).

**TABLE 1:** Intercellular mitochondrial transfer in macrophages under physiological conditions.

**Donor**	**Recipient**	**Transferred cargo(s)**	**Route**	**Transfer outcome**	**References**
Cardiomyocytes	Macrophages	Mitochondria; sarcomeric proteins	Exophers	Maintains homeostasis of cardiomyocytes	Nicolas-Avila et al., 2020
Adipocytes (WAT)	Macrophage subpopulation	Mitochondria	Internalization	Maintains systemic metabolic homeostasis	Brestoff et al., 2020

*WAT, white adipose tissue.*

**TABLE 2:** Intercellular mitochondrial transfer in macrophages under pathological conditions.

**Donor**	**Recipient**	**Pathological condition(s)**	**Induction factor(s)**	**Cargo**	**Route**	**Outcome**	**References**
BMSCs	AMs	Acute respiratory distress syndrome	LPS	Healthy mitochondria	EVs	Enhanced macrophage OXPHOS and phagocytosis to reduce inflammation and lung injury	Morrison et al., 2017
BMSCs	AMs	Sepsis; acute respiratory distress syndrome	*Escherichia coli*; LPS	Healthy mitochondria	TNTs	Enhanced macrophage bioenergetics and phagocytosis	Jackson et al., 2016; Jackson and Krasnodembskaya, 2017
BMSCs	BMMs	Oxidative stress	Culture expansion under 21% O_2_	Depolarized mitochondria; microRNAs	MVs	Outsourcing of mitophagy; inhibition of macrophage activation	Phinney et al., 2015
CMs	Macrophages	Cardiac stress	Isoproterenol	Dysfunctional mitochondria	Exophers	Enhanced transmitophagy	Nicolas-Avila et al., 2020
Adipocytes (WAT)	Macrophage subpopulation	Obesity; inflammation	High-fat diet; IFN-γ and LPS	Mitochondria	Internalization	Reduced transfers; accumulation of fat	Brestoff et al., 2020

*AM, alveolar macrophages; BMM, bone marrow derived macrophages; BMSC, bone marrow mesenchymal stem cell; CM, cardiomyocyte; EV, extracellular vesicle; IFN-γ, interferon-γ; LPS, lipopolysaccharide; MV, microvesicle; OXPHOS, oxidative phosphorylation; TNT, tunneling nanotube; and WAT, white adipose tissue.*

## Dynamic Regulation of Mitochondria Within and Between Cells

Mitochondria are double-membrane organelles that are extensively involved in cell functions. They are widely known as the cell’s “power house” because they generate adenosine triphosphate (ATP) via oxidative phosphorylation (OXPHOS) and host essential lipid metabolism pathways (Schon et al., 2012). The mitochondrial respiratory chain on the inner membrane of mitochondria converts the power of nicotinamide adenine dinucleotide and dihydroflavine-adenine dinucleotide from the Krebs cycle to an electrochemical proton gradient across the inner membrane (Scarpulla, 2008); this electrochemical gradient fuels ATP synthase to catalyze cellular ATP (Friedman and Nunnari, 2014). Byproducts of mitochondrial redox reactions include reactive oxidative species (ROS), which can initiate diverse cellular responses ranging from cell protection to mitochondrial fission and autophagy (Zorov et al., 2014). The electrochemical proton gradient also powers Ca^2+^ uptake through uniporters on the inner membrane to regulate cytoplasmic Ca^2+^ levels (De Stefani et al., 2011). As semiautonomous organelles, mitochondria contain mitochondrial DNA (mtDNA) and are capable of self-replication. MtDNA is a 16.5-kb circular double-stranded DNA that is highly compacted within the mitochondrial matrix and encodes the core proteins of the mitochondrial respiratory chain (Anderson et al., 1981; Friedman and Nunnari, 2014).

### Mitophagy: Mitochondrial Quantity and Quality Control

The integrity of mitochondria may be compromised owing to oxidative stress, starvation, ischemia–hypoxia, and aging (Spees et al., 2006; Gustafsson and Dorn, 2019; Liu et al., 2021), leading to energy exhaustion, ROS overproduction, and Ca^2+^-induced cell apoptosis (Bock and Tait, 2020). Mitophagy is an acute response to stress under changing developmental, bioenergetic, and environmental conditions that enable cells to meet the demands of metabolic reprogramming, mitochondrial quality control, and cell differentiation (Gustafsson and Dorn, 2019). Mitophagy is a process of cargo-specific autophagy that eliminates damaged mitochondria to regulate mitochondrial quality and quantity (Kim et al., 2007). During mitophagy, serine/threonine-protein kinase PINK1 is stabilized on the membranes of unwanted mitochondria for the subsequent recruitment of E3 ubiquitin-protein ligase parkin from the cytoplasm. Unwanted mitochondria then are marked by parkin-mediated ubiquitination in the outer mitochondrial membrane and recognized by autophagosomes (Pankiv et al., 2007; Matsuda et al., 2010; Okatsu et al., 2010). Other mitophagy pathways independent of ubiquitination are mediated by direct interaction between mitophagy receptors, including Bcl-2/adenovirus E1B 19-kDa protein-interacting protein 3-like (BINP3)/NIX, and several autophagosome proteins (Youle and Narendra, 2011; Zhang T. et al., 2016; Koentjoro et al., 2017).

### Mitochondrial Transfer

As well as mitophagy, mitochondria constantly undergo changes in their position and morphology to deal with stress and to meet the cell’s demands. Changes in intracellular position are driven by the attachment and movement of mitochondria along dynamic cytoskeletal tracks (Liesa et al., 2009; Rafelski, 2013). Changes in track arrangements, interactions between mitochondria and organelles including the endoplasmic reticulum and plasma membrane, and mitochondrial fission and fusion are the main factors that influence mitochondrial intracellular movements (Chen et al., 2005; Lackner et al., 2013; Rafelski, 2013). Changes in morphology most commonly involve fission and fusion dynamics. Fusion reverses the effects of stress on the cell by allowing functional mitochondria to complement dysfunctional ones, whereas fission can lead to cleansing of daughter mitochondria by mitophagy (Youle and van der Bliek, 2012).

Mitochondria movements are not constrained within cells but also take place between cells. Mitochondrial transfers occur both under physiological conditions, e.g., in tissue homeostasis and stemness maintenance and in pathological conditions such as hypoxia, inflammation, and cancer (Liu et al., 2021). The transferred cargos may contain either healthy or damaged mitochondria. Healthy mitochondria are transferred from donor cells to protect recipient cells from oxidative stress and apoptosis and to enhance their mitochondrial respiration. In the case of stroke, astrocytes release healthy mitochondria that enter neurons to promote ATP production and viability (Islam et al., 2012; Liu et al., 2014; Hsu et al., 2016). Transfers of healthy mitochondria also occur between cancer cells to promote their survival during chemotherapy (Lou et al., 2012; Desir et al., 2016; Zampieri et al., 2021). On the other hand, stressed cells can transfer damaged mitochondria to recipient cells to ease their burden of impaired mitochondria. This occurs between injured retinal ganglion cells and adjacent astrocytes and between acute leukemia T cells and bone-marrow-derived stem cells (Davis et al., 2014; Wang et al., 2018).

## Macrophage Derivation, Polarization, and Intercellular Communications

**FIGURE 1:**
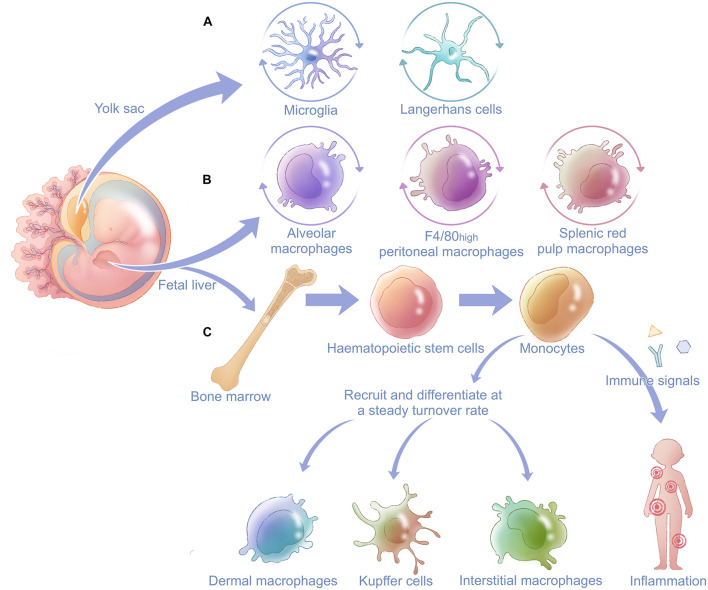
General derivation and distribution of macrophages in the body. Macrophages are derived from two main fetal organs: the fetal liver and yolk sac. **(A)** Microglia in the central neural system and Langerhans cells in epidermal tissue have prenatal origins from the yolk sac and renew themselves locally after being seeded in different systems. **(B)** Alveolar macrophages, F4/80^high^ peritoneal macrophages, and splenic red pulp macrophages originate from the fetal liver and also have postnatal self-renewal capacity. Fetal liver gives rise to hematopoietic stem cells in bone marrow. **(C)** Hematopoietic stem cells can develop into monocytes and finally differentiate into other tissue-resident macrophages such as dermal macrophages, Kupffer cells, and interstitial macrophages after birth. When stimulated by immune signals, monocytes can be recruited and differentiate into macrophages at inflammatory sites and innate immune.

Macrophages are highly plastic cells and are present in almost all tissues, as exemplified by alveolar macrophages (AMs) in lung, Kupffer cells in liver, Langerhans cells in epidermal tissue, osteoclasts in bone, splenic macrophages in spleen red pulp, F4/80^high^ peritoneal macrophages in peritoneum, and so on (Wynn et al., 2013). The classical definition of macrophages describes them as end cells of the mononuclear phagocytic lineage derived from circulating monocytes that originate in the bone marrow (Geissmann et al., 2010). However, more recent studies indicate heterogeneous origins of bone-marrow-derived macrophages (BMMs) in contrast to self-renewing embryo-derived ones, such as the yolk sac and fetal liver (Figure 1; Ginhoux et al., 2010; Hoeffel et al., 2012; Schulz et al., 2012). Many tissues contain both local self-renewing and peripheral monocyte-derived populations of macrophages (Schulz et al., 2012).

### Macrophage Polarization and Function

**FIGURE 2:**
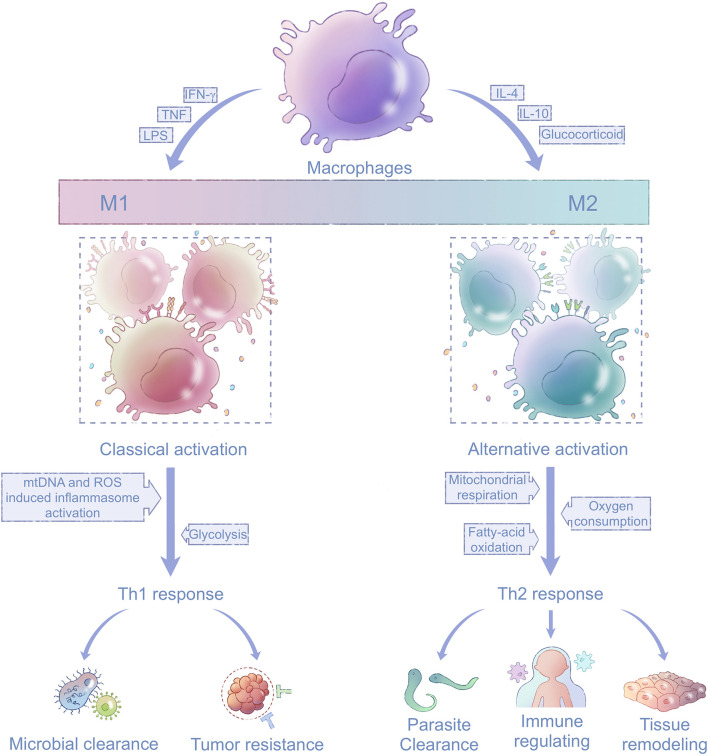
Mitochondria in macrophage polarization. Macrophages are activated and polarized in response to different stimuli, including TNF, IFN-γ, LPS, IL-4, IL-10, and glucocorticoids. The states of macrophages fall between two extremes, M1 and M2 phenotypes, which participate in the Th1 and Th2 immune responses, respectively. Energy metabolism in M1-polarized macrophages shifts to glycolysis compared with their precursors, and M1-polarized macrophages are activated by mtDNA and mitochondria-produced ROS (left). Energy metabolism in M2-polarized macrophages mainly depends on mitochondrial respiration fueled by oxygen and fatty acids (right). TNF, tumor necrosis factor; IFN-γ, interferon-γ; LPS, lipopolysaccharide; IL-4, interleukin-4; IL-10, interleukin-10; ROS, reactive oxidative species; and mtDNA, mitochondrial DNA.

Macrophages have two main activation states: M1 and M2 polarization (Figure 2; Locati et al., 2020). M1-polarized macrophages are activated by interferon-γ (IFN-γ), lipopolysaccharide (LPS), granulocyte-macrophage colony-stimulating factor, or tumor necrosis factor (TNF; Biswas and Mantovani, 2010). The toll-like receptor (TLR) for LPS and receptors for cytokines such as IFN-γ are thus activated and induce subsequent expression of transcription factors nuclear factor kappa-B (NF-κB), interferon regulatory factor 3 (IRF-3), and signal transducer and activator of transcription 1 (STAT1; Sengupta et al., 1996; Sica and Bronte, 2007). These transcription factors are transported into the nucleus, where they upregulate genes related to M1-polarized macrophages (Taniguchi et al., 2001; Gao et al., 2018). M1-polarized macrophages exhibit enhanced phagocytosis mediated by increased secretion of pro-inflammatory cytokines and chemotactic factors; thus, they facilitate the removal of non-self components (Sica and Mantovani, 2012) and play important parts in Th1-mediated immune responses (Biswas and Mantovani, 2010). M2-polarized macrophages are stimulated by interleukin-4 (IL-4) or interleukin-10 (IL-10) signaling, which induces signal transducer and activator of transcription 6 (STAT6), interferon regulatory factor 4 (IRF-4), and peroxisome proliferator-activated receptor γ (PPARγ; Odegaard et al., 2007; Czimmerer et al., 2018). M2-polarized macrophages can be further divided into M2a, M2b, and M2c subgroups. The M2a and M2b phenotypes are activated by IL-4 and promote an immune response mediated by Th2 (Stein et al., 1992). By contrast, M2c inhibits the immune response and favors tissue remodeling after activation by IL-10 or glucocorticoids (Curtale et al., 2013, 2017).

### Mitochondria in Macrophage Polarization and Signal Transduction

Macrophages undergo mitochondria-related metabolic reprogramming during activation (Haschemi et al., 2012; Huang et al., 2014; Jin et al., 2014). M1-polarized macrophages depend mainly on glycolysis as the first line of defense, whereas M2-polarized macrophages largely rely on oxygen consumption by mitochondrial respiration for their long-term functions (Haschemi et al., 2012; Galvan-Pena and O’Neill, 2014). Increased glucose utilization in IL-4-stimulated macrophages requires activation of the mechanistic/mammalian target of rapamycin complex 2 pathway, which operates in parallel with the IL-4Rα-STAT6 pathway to facilitate M2 activation via induction of IRF-4 (Huang et al., 2016). PPARγ-coactivator-1b (PGC-1b) induces mitochondrial biogenesis and is also indispensable for M2 polarization (Vats et al., 2006), and cell-autonomous lysosomal-based lipolysis and fatty-acid oxidation fuel the mitochondrial metabolism to maintain the M2 phenotype (Huang et al., 2014).

In addition to metabolism alteration, mitochondrial damage-associated molecular patterns (DAMPs) such as mtDNA and byproducts of mitochondrial respiration such as ROS have important roles in the initiation and transduction of signals in the immune response, especially in M1 activation (Nakahira et al., 2011; Zhou et al., 2011). The synthesis of mtDNA, which is induced after the engagement of TLRs, is crucial for NACHT and leucine-rich repeat protein 3 (NLRP3) signaling in M1-polarized macrophages; dysregulated NLRP3 inflammasome activity results in uncontrolled inflammation (Zhong et al., 2018). ROS are essential bactericidal components generated primarily via the phagosomal NADPH oxidase machinery by phagocytes including macrophages (Lambeth, 2004; West et al., 2011). ROS promote production of pro-inflammatory cytokines in response to LPS via decreasing the dephosphorylation of mitogen-activated protein kinases (MAPKs) including c-Jun N-terminal kinase, extracellular signal-regulated kinase, and p38 MAPK phosphorylation (Bulua et al., 2011). ROS also contribute to NLRP3 inflammasome activation (Sorbara and Girardin, 2011). Moreover, mitochondrial ROS are critical to the differentiation of M2-polarized macrophages (Angajala et al., 1605). In a study of TAMs, which are similar to M2-polarized macrophages in terms of their pro-angiogenic and immune-suppressive functions, inhibition of superoxide production was shown to specifically block the differentiation of M2 macrophages (Zhang et al., 2013). However, the inhibitory effect of ROS elimination on macrophage differentiation was overcome when macrophages were polarized to the M1 phenotype (Zhang et al., 2013).

### Cell–Cell Communications Between Macrophages and Surrounding Cells

Cell–cell communications occur frequently between macrophages and adjacent tissue cells. Macrophages integrate into host tissues; this entails their specialization in response to the local environment (Varol et al., 2015). Local cells imprint the specific functions of macrophages (Varol et al., 2015), as exemplified by AMs, osteoclasts, and microglia. Alveolar epithelial cells are a major source of colony-stimulating factor 2 (CSF-2), which is necessary for the differentiation of AMs (Guilliams et al., 2013). The differentiation and function of osteoclasts are regulated by the balance of receptor activator of NF-κB ligand (RANKL) and osteoprotegerin produced by osteoblasts (Takayanagi et al., 2002; Boyle et al., 2003; Ikebuchi et al., 2018). Molecules of neuronal origin control microglial motility and functions via chemotaxis, neurotransmitters, and purinergic and adenosine signaling pathways (Crain et al., 2009; Mead et al., 2012; Limatola and Ransohoff, 2014).

**FIGURE 3:**
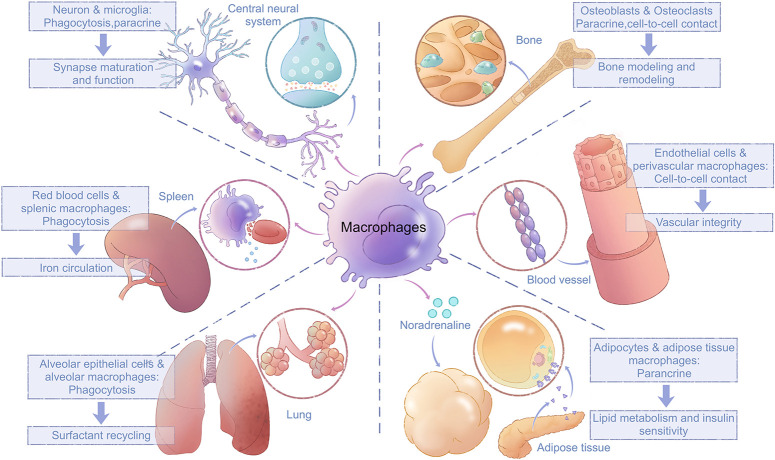
Interactions between macrophages and tissue cells. Macrophages interact closely with surrounding tissue cells through paracrine mechanisms, cell-to-cell contacts, and direct phagocytosis; these interactions are vital for tissue development and their normal function. Microglia trim synapses and release signal molecules to promote neural synapse maturation and function. Splenic macrophages phagocytose red blood cells and facilitate iron circulation. Alveolar macrophages phagocytose surfactants produced by alveolar epithelial cells. Bone-resident osteoclasts interact with osteoblasts through both cell-to-cell contacts and paracrine communication. Perivascular macrophages maintain vascular integrity by attenuating phosphorylation of VE-cadherin in endothelial cells via cell-to-cell contact. Macrophages in white adipose tissue regulate lipid metabolism and insulin sensitivity in adipocytes via paracrine effects of noradrenalin. VE, vascular endothelial.

Cell–cell communications between macrophages and tissue cells also facilitate tissue-specific functions of macrophages and contribute to the development and specific functions of resident tissues (Figure 3). The most direct type of cell–cell communication is based on the prototypical macrophage function, phagocytosis (Nagata, 2018). For example, macrophages in spleen red pulp phagocytose red blood cells (RBCs) to facilitate iron circulation (Rodrigues et al., 2017). Slight modifications of the cell membranes of RBCs, such as those associated with RBC senescence or damage, are sensed by macrophages, which phagocytose such RBCs and return iron to erythroid progenitors (Korolnek and Hamza, 2015). Microglia (macrophages in the central neural system) contribute to neural synapse maturation and brain development by synaptic pruning (Paolicelli et al., 2011).

In addition to phagocytosis, other forms of cell–cell communication exist between macrophages and their surrounding cells. Microglia can also release signaling molecules including brain-derived neurotrophic factor and microvesicles (MVs) containing cytosolic proteins, lipids, and microRNAs to regulate synaptic activity (Parkhurst et al., 2013; Maas et al., 2017). Macrophages in adipose tissues regulate lipid metabolism and insulin sensitivity in adipocytes via paracrine effects of noradrenalin (Pirzgalska et al., 2017; Flaherty et al., 2019). Perivascular macrophages in capillaries attenuate vascular endothelial-cadherin phosphorylation in endothelial cells to limit blood vessel permeability and maintain vascular integrity (He et al., 2016; Lapenna et al., 2018). BMMs develop into osteoclasts, where they coordinate with osteoblasts for bone modeling and remodeling through both cell contacts and ligand–receptor interactions (Boyle et al., 2003). The best-studied such mutual interactions are RANKL signaling in osteoclasts and its reverse signaling in osteoblasts, which maintains the balance between osteoclast maturation and function (Takayanagi et al., 2002; Ikebuchi et al., 2018). After osteoclast maturation, the proton pump in osteoclasts acidifies the resorption organelle and releases lytic enzymes to realize bone resorption (Boyle et al., 2003). In general, macrophages and tissue cells interact both directly and indirectly to maintain physiological functions of tissues.

## Macrophage-Related Mitochondrial Transfer

**FIGURE 4:**
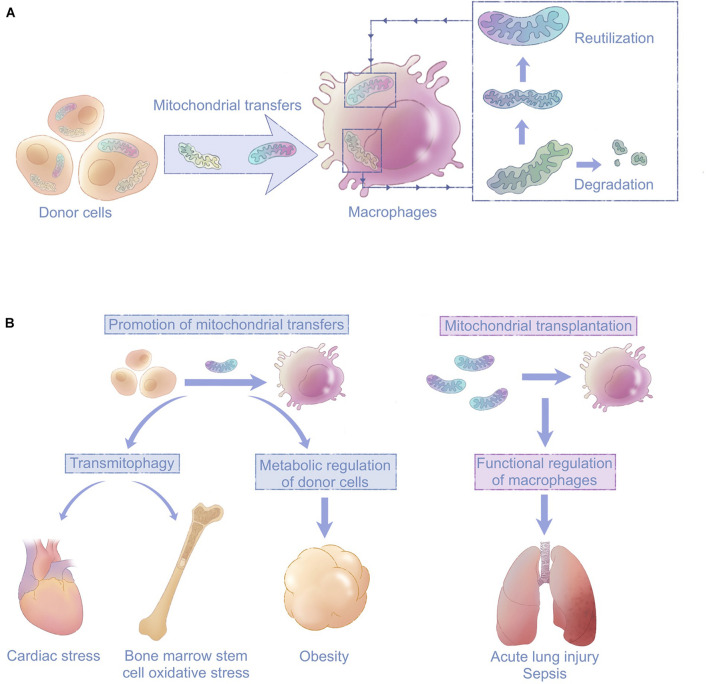
Macrophages are emerging as key players in mitochondrial transfers with clinical implications. **(A)** Macrophages mediate intercellular mitochondrial transfers of either healthy or damaged mitochondria from surrounding tissue cells. Transferred healthy mitochondria improve the function of recipient macrophages. Damaged mitochondria are degraded via mitophagy to ease donor cells’ stress or reutilized in macrophages. **(B)** Mitochondrial transfers involving macrophages can be targeted for therapeutic purposes through promotion of mitochondrial transfers or mitochondrial transplantation; this is a promising approach for diseases including cardiac stress, obesity, acute lung injury, and sepsis.

The involvement of macrophages in intracellular mitochondrial transfers is emerging as a critical phenomenon in various tissues. Macrophages often function as recipients that digest depolarized or fragmented mitochondria, thereby favoring the survival and maintaining the functions of surrounding cells. Transfers of healthy mitochondria also contribute to the polarization and homeostasis of both recipient and donor macrophages. In general, macrophage-related mitochondrial transfers have mutual effects on both macrophages and their surrounding cells (Figure 4A).

### Macrophages Mediate Transmitophagy

Damaged mitochondria are generally degraded by mitophagy, a cell-autonomous activity (Davis and Marsh-Armstrong, 2014). In the case of ganglion cells and astrocytes, transcellular mitophagy (hereafter called transmitophagy) has been proposed as a means of extracellular degradation that lightens the burden of stressed cells (Davis et al., 2014). Recently, different groups of macrophages have been reported as potential handlers of damaged mitochondria during transmitophagy. Accumulating evidence also indicates that the mitophagy level inside macrophages influences macrophage polarization and function (Table 3; Kim et al., 2016; Larson-Casey et al., 2016; Zhao et al., 2017; Bhatia et al., 2019; Patoli et al., 2020; Zhang et al., 2020). Thus, transmitophagy may exert influences on both donor cells and recipient macrophages to regulate tissue homeostasis.

**TABLE 3:** Mechanisms of mitophagy in macrophages.

**Cell type**	**Pathological condition**	**Mechanism**	**Outcome**	**References**
SM	Atherosclerosis	Inhibition of mitophagy via mTORC1 signaling	Macrophage apoptosis	Zhang et al., 2020
SM	Polymicrobial sepsis	Inhibition of PINK1-dependent mitophagy through activation of inflammatory caspases 1 and 11	Macrophage activation	Patoli et al., 2020
BMM	Kidney fibrosis	Inhibition of mitophagy through downregulation of MFN2 and parkin	Promotion of M2 polarization	Bhatia et al., 2019
RM	Diabetic nephropathy	Inhibition of mitophagy	Promotion of M1 polarization	Zhao et al., 2017
AM	Idiopathic pulmonary fibrosis	Promotion of mitophagy via enhancing mitochondrial ROS production	Macrophage survival	Larson-Casey et al., 2016
BMM	Sepsis	Promotion of mitophagy induced by SESN2	Suppression of inflammasome hyperactivation in macrophages	Kim et al., 2016

*AM, alveolar macrophages; BMM, bone marrow derived macrophages; RM, renal macrophages; and SM, splenic macrophage.*

#### Cardiomyocytes Transfer Mitochondria to Macrophages

Cardiomyocytes (CMs) are highly specialized smooth muscle cells with extremely long lifespan and low turnover rate, which rely on a large pool of mitochondria to meet their intensive energy demands (Bergmann et al., 2009). Accumulation of damaged mitochondria leads to cardiac hypertrophy and heart failure (Bertero and Maack, 2018). Thus, elimination of dysfunctional mitochondria is vital for CM survival and function. Although most studies of CMs have focused on their intracellular clearance of fragmented mitochondria (Chen and Dorn, 2013; Gong et al., 2015; Tong et al., 2019), Nicolas-Avila et al. (2020) recently proposed intercellular mitochondrial transfer between CMs and surrounding cardiac-resident macrophages (cMACs) as an extracellular route by which CMs dispose of dysfunctional mitochondria.

In this study, altered mitochondrial morphology and reduced cristae density were observed in cMAC-deficient mice, suggesting impaired cardiac mitochondria homeostasis. Moreover, cMAC-deficient mice showed impaired systolic function, which could be restored by supplementation with cMACs; this finding emphasized the crucial role of cMACs in ensuring the mitochondria health of CMs (Nicolas-Avila et al., 2020). Further investigation revealed that cMACs took up mitochondria with compromised membrane integrity from CMs via exophers under physiological conditions (Nicolas-Avila et al., 2020). Under pathological conditions, as in an isoproterenol-induced mouse model of cardiac ischemia, dysfunctional mitochondrial transfer from CMs to cMACs was significantly increased. These results imply that the outsourcing of dysfunctional mitochondria from CMs to cMACs might have a protective function against cardiac stress (Nicolas-Avila et al., 2020). Nevertheless, as mitophagy and mitochondria influence the activation state of cMACs, how cMACs endure damaged mitochondria from CMs under stress remains unknown, as does how stress itself influences ability of cMACs to deal with dysfunctional mitochondria. Moreover, considering the heterogeneity of macrophages, single-cell sequencing may help to identify distinct subgroups responsible for transmitophagy.

#### Bone Marrow Stem Cells Transfer Mitochondria to Macrophages

Bone marrow mesenchymal stem cells (BMSCs) naturally reside in a hypoxic stem cell niche and regulate self-renewal and mobilization of hematopoietic stem cells via crosstalk with adjacent macrophages (Mendez-Ferrer et al., 2010; Chow et al., 2013; Morrison and Scadden, 2014). In an *ex vivo* co-culture system, macrophages were shown to receive depolarized mitochondria from BMSC exosomes, thus enhancing the ability of BMSCs to deal with oxidative stress by improving mitochondrial bioenergetics. Depolarized mitochondria are first loaded into LC3-positive vesicles and then migrate toward the cell periphery, where they are incorporated into outward budding blebs and subsequently taken up by macrophages (Phinney et al., 2015). This phenomenon may also occur *in vivo* between local macrophages and BMSCs administered to patients. BMSC-to-macrophage mitochondrial transfers represent a possible mechanism by which macrophages in stem cell niches protect BMSCs under stress, yet direct *in vivo* evidence for this mechanism is still lacking (Phinney et al., 2015).

### Metabolic Regulation of Donor Cells

As mitochondria are centers of energy metabolism and coordinate aerobic respiration fueled by either glucose or lipids (Schon et al., 2012), mitochondrial transfers involving macrophages are not only used for transmitophagy but also meet metabolic ends, as exemplified by adipocytes in white adipose tissue (WAT) and adipose tissue macrophages (ATMs). In a recent study focusing on mitochondrial transfers between adipocytes in WAT and ATMs, nearly half of the ATMs in mice internalized mitochondria from neighboring adipocytes under physiological conditions (Brestoff et al., 2020). The significance of these mitochondrial transfers was confirmed in a mouse model of obesity fed a high-fat diet, where adipocyte-to-macrophage mitochondrial transfers were drastically decreased (Brestoff et al., 2020).

Adipose tissue macrophages regulate the glucose utilization and energy expenditure of adipocytes (Pirzgalska et al., 2017; Flaherty et al., 2019), and obesity induces a phenotype switch in ATMs (Lumeng et al., 2007). In turn, changes in ATM phenotypes contribute to WAT inflammation and obesity-induced insulin resistance (Han et al., 2013; Kratz et al., 2014). Indeed, decreased adipocyte-to-macrophage mitochondrial transfers are related to an obesity-induced inflammatory state in WAT. First, the pro-inflammatory environment induced by IFN-γ, LPS activation, and M1 polarization contributes to a macrophage-intrinsic impairment in mitochondrial uptake (Brestoff et al., 2020). Second, the uptake ability of adipose-resident macrophages depends on the heparan sulfate biosynthesis pathway, which has been reported to show anti-inflammatory effects (Brestoff et al., 2020). Inhibition of heparan sulfate biosynthesis leads to aberrant mitochondrial uptake, accompanied by decreased energy expenditure and fat accumulation in adipose tissue (Brestoff et al., 2020).

Intriguingly, gene enrichment analysis of mitochondria-recipient macrophages has defined a transcriptionally distinct subgroup of macrophages that are capable of mitochondrial internalization, with traits resembling those of anti-inflammatory macrophages (Brestoff et al., 2020). More investigations are needed to trace the derivation and characteristics of the transcriptionally distinct macrophage subgroups, as well as their response to the metabolic state in obesity, in order to understand the complexity of ATMs. Moreover, as evidence suggests that ATMs are highly plastic according to their surrounding environment, and obesity is strongly associated with the number, derivation, and functional changes of ATMs (Lumeng et al., 2008; Xu et al., 2013), regulation of ATM subgroups toward mitochondrial uptake subgroups is a promising therapeutic approach for obesity and related metabolic syndromes.

### Functional Regulation of Macrophages by Mitochondrial Transfers

In addition to the favorable effects on surrounding cells of mitochondrial transfers from macrophages, macrophages themselves are influenced by transferred mitochondria. BMSCs modulate the function of AMs by transferring mitochondria via both contact-dependent and paracrine routes; this modulation can be exploited as a mechanism for BMSC therapy for acute lung injury (Jackson et al., 2016; Morrison et al., 2017). In *Escherichia coli*-treated co-culture system, MSCs were shown to transfer mitochondria to human macrophages via tunneling nanotubes (TNTs); this enhanced their phagocytic capacity and facilitated the antimicrobial effects of the BMSCs (Jackson et al., 2016). In a later study using a transwell system for macrophage and BMSC co-culture, BMSCs significantly increased the proportion of M2-polarized and phagocytic macrophages via transferring BMSC-derived extracellular vesicles (EVs) containing healthy mitochondria (Morrison et al., 2017).

In murine models, adoptive transfer of murine AMs treated with MSC-derived EVs protects mice from LPS-induced lung injury by alleviating the inflammatory cell recruitment, suggesting that anti-inflammatory M2 polarization occurs when AMs receive healthy mitochondria (Morrison et al., 2017). Importantly, BMSC-conditioned medium taken from rhodamine-6G-pretreated MSCs with dysfunctional mitochondria could not cause such changes, indicating the presence of functional mitochondria rather than mitochondrial components that induce such activation changes (Morrison et al., 2017).

Instead of mitochondria-derived immune signals, an increased energy supply from functional mitochondria is responsible for enhanced phagocytosis in AMs; the ATPase inhibitor oligomycin completely reversed the effect of MSC-conditioned medium on BMM phagocytosis (Morrison et al., 2017). However, AMs are a heterogeneous group of cells, especially in acute lung injury, which is characterized by acute immune response and subsequent tissue repair (Duan et al., 2012; Short et al., 2014). Whether self-replicative tissue-resident AMs or monocyte-derived AMs recruited during the acute immune response enable mitochondrial recipients to undergo M2 polarization and change of function remains unclear. In addition, mitochondrial transfers enhance the function of recipient macrophages by improving their mitochondrial bioenergetics (Phinney et al., 2015). Unhealthy mitochondria extruded by BMSCs still exhibit residual membrane potential, which provides evidence for their mitochondrial membrane integrity and fusion ability (Phinney et al., 2015). Depolarized but not totally fragmented mitochondria undergo mitochondrial fusion in macrophages for reutilization (Phinney et al., 2015). In addition to mitochondria, these vesicles contain miR451, miR1202, miR630, and miR638, which represses TLR expression, thereby tolerizing macrophages to mitochondrial-transfer-induced inflammation caused by excessive mtDNA (Phinney et al., 2015).

## How Macrophages Mediate Mitochondrial Transfers

Macrophages mediate mitochondrial transfers through various mechanisms in different systems. Phagocytosis, the most typical macrophage function, contributes substantially to the mediation of mitochondrial transfers by macrophages (Phinney et al., 2015; Nicolas-Avila et al., 2020). In an *ex vivo* oxidative stress model of BMSCs, BMMs nibbled the surfaces of human BMSCs, enabling uptake of mitochondria-containing phagosomes budding from the plasma membrane (Phinney et al., 2015). Pre-incubation with dextran sulfate, an inhibitor of phagocytosis, significantly reduced uptake of MVs from BMSCs by BMMs (Phinney et al., 2015). Isolated cMACs feature large phagolysosome-like vacuoles and have been shown to actively phagocytose materials from CMs; these materials were later proven to be mitochondria-contained exophers (Nicolas-Avila et al., 2020). The engulfed exophers become Lamp1^+^ phagolysosomes in cMACs (Nicolas-Avila et al., 2020). Western blotting analyses revealed inflammasome activation in the hearts of cMAC-depleted mice, possibly owing to the presence of free mitochondria and mtDNA caused by abrogated mitochondria transfers in the absence of cMACs (Oka et al., 2012; Nicolas-Avila et al., 2020). Inflammasome activation in turn caused autophagic arrest and impaired exopher production in CMs (Nicolas-Avila et al., 2020). Therefore, cMACs prevent inflammasome activation and protect autophagy flux in CMs to support exopher formation for mitochondrial transfers (Nicolas-Avila et al., 2020).

Whether macrophages obtain mitochondria through cell–cell contact, such as through TNTs, in addition to phagocytosis was also investigated in the case of BMSC antimicrobial therapy (Jackson et al., 2016). Depletion of AMs abrogates the antimicrobial effects of BMSCs (Jackson et al., 2016), and TNTs containing mitochondria are extended from BMSCs to AMs (Jackson et al., 2016). It seems that BMSCs play an active part in this case. However, mitochondrial transfers were reduced but still evident after blockage of TNT formation in BMSCs by cytochalasin B (Jackson et al., 2016); in a later study, this was attributed to AMs also acquiring BMSC mitochondria through EVs in a manner independent of TNT formation by BMSCs (Islam et al., 2012). Therefore, although TNTs formed by BMSCs are partially responsible for the acquisition of mitochondria by AMs, the AMs also acquire mitochondria from BMSCs via EVs (Islam et al., 2012; Jackson et al., 2016).

Furthermore, several critical molecules that may contribute to macrophage-mediated mitochondrial transfer have been identified. For example, AMs selectively uptake mitochondria containing EVs from BMSCs by recognizing CD44 on the surfaces of EVs (Islam et al., 2012). Anti-CD44 antibody partially abrogated the effects of BMSC-conditioned medium on macrophages, whereas antibodies administered to AMs in the absence of MSC-conditioned medium had no influence (Islam et al., 2012). In addition, EXT1, an important gene in the heparan sulfate biosynthesis pathway, has been reported to be indispensable for ATMs to obtain mitochondria from adipocytes (Brestoff et al., 2020). Conditional deletion of EXT1 in myeloid cells reduces heparan sulfate levels in ATMs, impairs mitochondria transfer, and promotes fat mass accumulation (Brestoff et al., 2020). However, the direct association between the heparan sulfate biosynthesis pathway and the function of macrophages remains unclear (Brestoff et al., 2020). More studies are needed to decipher the mechanisms underlying macrophage-mediated mitochondrial transfers.

## Targeting Mitochondria in Macrophages for Therapeutic Purposes

Great progress has been made in recent years in modulating the tissue environment via macrophages, particularly in the field of antitumor immunotherapy (Tacke, 2017; Xia et al., 2020). Most of those therapies target molecular pathways related to the recruitment and phenotypes of macrophages (Pathria et al., 2019), for instance, the CSF-1 receptor (Cassier et al., 2015; Cannarile et al., 2017) and agonistic CD40 therapy (Wiehagen et al., 2017). Targeting energy metabolism and mitochondria-related signal transduction in macrophages shows good prospects for developing efficient interventions.

Targeting glycolysis and mitochondrial ROS have been reported as effective therapeutic strategies for controlling inflammation mediated by M1-polarized macrophages. Dimethyl fumarate, a derivative of Krebs cycle intermediate fumarate, downregulates aerobic glycolysis in activated peritoneal macrophages to inhibit inflammation; thus, it has a critical role in the treatment of multiple sclerosis (Linker and Haghikia, 2016). Similarly, itaconate, an endogenous metabolite, is required for activation of the anti-inflammatory transcription factor Nrf2 in LPS-activated mouse and human macrophages (Mills et al., 2018). In addition, 4-octyl itaconate, a cell-permeable itaconate derivative, protects against LPS-induced cytokine production and inflammation *in vivo* (Mills et al., 2018). On the other hand, diphenyliodonium, a global and mitochondrial ROS scavenger, was shown to impair LPS-induced NLRP3 expression, thereby inhibiting IL-1β and IL-18 production in macrophages (Sazanov, 2007). Other promising candidates include metformin and rotenone, which regulate glycolysis and ROS via targeting pyruvate kinase and could also inhibit inflammation induced by M1-polarized macrophages (Palsson-McDermott et al., 2015; Mills and O’Neill, 2016; Peruzzotti-Jametti and Pluchino, 2018). Activation of M2-polarized macrophages can be regulated by OXPHOS. Acute inhibition of the polyamine-eIF5A-hypusine axis by Eif5a small interfering RNA (siRNA), Dhps-siRNA, and deoxyhypusine synthase inhibitor GC7 blunts OXPHOS-dependent M2 activation while leaving aerobic glycolysis-dependent M1 activation intact (Puleston et al., 2019). Genetic and GC7-driven inhibition of eIF5AH silenced mitochondria has been reported to prevent anoxic death of kidney cells and to improve outcomes of kidney transplants (Melis et al., 2017).

In addition to conventional M1/M2 polarization, high levels of fatty acid oxidation in TAMs promote mitochondrial OXPHOS, ROS production, and JAK1 phosphorylation, leading to STAT6 activation and transcription of genes that regulate TAM generation and function (Su et al., 2020). Given the importance of fatty acid oxidation in TAMs, interfering in lipid metabolism could be a promising therapeutic approach for cancer (Su et al., 2020). In atherosclerosis, Dicer plays a protective part in coordinately regulating the inflammatory response in lesional macrophages through enhancing fatty-acid-fueled mitochondrial respiration. Promoting Dicer/miR-10a-dependent metabolic reprogramming in macrophages has potential therapeutic applications for the prevention of atherosclerosis (Wei et al., 2018).

Studies of mitochondrial transfer provide new strategies for modification of macrophages. In the cases of the myocardium and adipose, where spontaneous cell-to-macrophage mitochondrial transfers occur under physiological conditions (Brestoff et al., 2020; Nicolas-Avila et al., 2020), impaired ability of macrophages to take up mitochondria from surrounding cells could contribute to pathogenesis. Enhancing macrophage-related mitochondrial transfer is thus a promising therapeutic strategy. In acute lung injury and sepsis (Islam et al., 2012; Jackson et al., 2016), mitochondrial transplantation into macrophages could promote macrophage phagocytosis and train the immune system (Figure 4B; Yamada et al., 2020). The development of new treatments calls for more studies on the basic mechanisms underlying mitochondrial transfers involving macrophages. Key molecules responsible for mitochondrial transfer signaling in macrophages and mitochondrial transfer routes should be identified.

## Conclusion and Perspectives

Mitochondrial transfers from donor cells promote the survival of recipient cells by enabling the recovery of mitochondrial function, as exemplified by neurons and osteocytes with low self-renewal rates (Hayakawa et al., 2016; Gao et al., 2019), chemoresistant cancer cells (Pasquier et al., 2013; Tan et al., 2015; Moschoi et al., 2016), and therapeutic use of stem cells (Zhang Y. L. et al., 2016; Yao et al., 2018). Recent studies have reported that macrophage-related mitochondrial transfers have important roles in processing unhealthy mitochondria as well as utilizing healthy mitochondria. Notably, mitochondria transferred from tissue cells to macrophages could also function as important messengers. In acute inflammation, activated monocytes give out mitochondria-related DAMPs including mitochondrial membrane components and mitochondrial 16S ribosomal RNA to activate an inflammatory response in endothelial cells (Pober and Sessa, 2007; Ait-Oufella et al., 2010; Puhm et al., 2019). Macrophages, which are important immune cells, can also be activated by mitochondria-related DAMPs (Nakahira et al., 2011; Zhou et al., 2011); therefore, mitochondrial transfers could function as immune signals. Besides, since macrophages in acute inflammatory responses are derived from recruited monocytes (Geissmann et al., 2010), macrophages might play a similar role as monocytes to give out mitochondrial components as immune signals.

Unhealthy mitochondria received by macrophages usually undergo either reutilization or degradation (Phinney et al., 2015; Brestoff et al., 2020; Nicolas-Avila et al., 2020). Reutilization of unhealthy mitochondria in macrophages resident in the stem cell niche is achieved by mitochondrial fusion to enhance OXPHOS in these macrophages (Phinney et al., 2015). Alternatively, degradation of unhealthy mitochondria can be achieved by transmitophagy in macrophages (Phinney et al., 2015; Brestoff et al., 2020; Nicolas-Avila et al., 2020). In addition, unhealthy mitochondria received by macrophages may be extruded by migrasomes, which are newly identified vesicular structures that discharge cellular contents during migration (Ma et al., 2015). In an *ex vivo* study, BMMs exposed to mild mitochondria stress induced by carbonyl cyanide 3-chlorophenylhydrazone were observed to leave behind migrasomes containing damaged mitochondria (Jiao et al., 2021), indicating that BMMs may be donor cells for mitochondrial transfers (Jiao et al., 2021). Further studies are required to determine whether migrasomes containing mitochondria are received by surrounding cells as a route of mitochondrial transfer.

Macrophages are widely distributed, enabling them to maintain tissue homeostasis, and related to various diseases (Wynn et al., 2013). However, studies to date have only described limited situations in which macrophage-related transfers exert their effects. This is the beginning of a conversation, not the final word. Targeting mitochondrial transfers in macrophages is a strategy that shows great potential in a range of fields including cancer and infectious diseases (Na et al., 2018). Therefore, future studies should focus on the development of techniques to regulate macrophage-related mitochondrial transfers for therapeutic purposes (Figure 4B).

## Author Contributions

YP and JG provided the essential ideas for this work and performed the literature search and drafted the article. CZ and JG critically revised the manuscript. All authors contributed to the article and approved the submitted version.

## Conflict of Interest

The authors declare that the research was conducted in the absence of any commercial or financial relationships that could be construed as a potential conflict of interest.

## Publisher’s Note

All claims expressed in this article are solely those of the authors and do not necessarily represent those of their affiliated organizations, or those of the publisher, the editors and the reviewers. Any product that may be evaluated in this article, or claim that may be made by its manufacturer, is not guaranteed or endorsed by the publisher.

## Abbreviations

AM: alveolar macrophage
ATM: adipose tissue macrophage
BMM: bone-marrow-derived macrophages
BNIP-3: Bcl-2/adenovirus E1B 19 kDa interacting with protein-3
BMSC: bone marrow mesenchymal stem cell
CCCP: carbonyl cyanide 3-chlorophenylhydrazone
CM: cardiomyocyte
cMAC: cardiac-resident macrophage
DAMP: damage-associated molecular pattern
*E. coli*: *Escherichia coli*
FADH2: dihydroflavine-adenine dinucleotide
GM-CSF: granulocyte-macrophage colony-stimulating factor
IFN- γ: interferon- γ
IL-4: interleukin-4
IL-10: interleukin-10
LPS: lipopolysaccharide
mtDNA: mitochondrial DNA
MV: microvesicle
NADH: nicotinamide adenine dinucleotide
OXPHOS: oxidative phosphorylation
RM: renal macrophages
ROS: reactive oxidative species
SM: splenic macrophages
TAM: tumor associated macrophages
TLR: toll-like receptor
TNF: tumor necrosis factor
TNT: tunneling nanotube
WAT: white adipose tissue.
